# Histologically Malignant Solitary Fibrous Tumour of the Anterior Thoracic Wall: A Case Report and Review of the Literature

**DOI:** 10.1155/2010/257167

**Published:** 2010-06-20

**Authors:** Maria Archontaki, Dimitris P. Korkolis, Niki Arnogiannaki, Stelios Hatzijiannis, Panagiotis Dendrinos, Christos Megapanos, Dimitris Kassotakis, Georgios Kokkalis

**Affiliations:** ^1^Department of Plastic and Reconstructive Surgery, Greek Anticancer Institute, St. Savvas Hospital, 171 Alexandras Avenue, 115 22 Athens, Greece; ^2^Department of Surgery, Greek Anticancer Institute, St. Savvas Hospital, 171 Alexandras Avenue, 115 22 Athens, Greece; ^3^Department of Pathology, Greek Anticancer Institute, St. Savvas Hospital, 171 Alexandras Avenue, 115 22 Athens, Greece

## Abstract

Solitary fibrous tumour (SFT) is a rare oncological entity that most often arises in the pleura. Over the past 10 years, the tumour has been described at numerous extrapleural locations. We present the case of a 42-year-old female Caucasian patient with an extrapleural SFT located at the anterior thoracic wall for 22 years, with atypical histological characteristics and clinical features of malignancy. Management consisted of a wide surgical resection, plastic reconstruction, and postoperative radiotherapy. Although extrapleural SFT usually behaves as a benign soft tissue tumour, it can also present with a more aggressive local behavior, including locoregional recurrence or metastasis. In that case, a multidisciplinary approach is required for accurate diagnosis and proper management.

## 1. Introduction

Extrapleural SFTs account for 0.6% of all soft tissue tumours [1]. Malignant extrapleural SFT is an even more rare neoplasm and has been described only in limited series [2, 3] and case reports [4–6]. In a large case series of SFTs, Gold et al. [1] reported that no difference exists between intrathoracic SFT and extrapleural SFT regarding rates of malignant pathological features but extrapleural SFTs had a significantly higher rate of locoregional recurrence suggesting a more aggressive clinical behavior. In a more recent study of Cranshaw et al. [7] 55% of extrapleural SFTs of this series showed malignant features. 

We hereby present the unusual case of a 42-year-old patient presented with a large, histologically malignant SFT of the anterior thoracic wall.

## 2. Case Report

A 42-year-old female, Caucasian, patient was referred to our institute suffering from a large, pedicled, painless, slow-growing mass, located on the anterior thoracic wall. In addition the patient was complaining of fatigue and weakness for the past 3 months. The lesion developed as a long-standing tumour with duration of approximately 22 years. There was a history of incomplete previous resection of the tumour twice in the past, resulting in locoregional recurrence. The tumour was diagnosed according to the first histological report as a dermatofibrosarcoma protuberance (DFSP) and according to the second one as a hemangiopericytoma. In addition to recurrence, neglect on the part of patient, patient's fear, and embarrassment for her disease may have played a role in the development of this chronic large tumour. 

**Figure 1:**
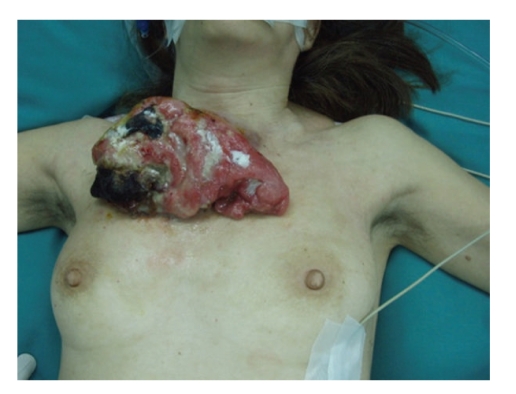
Solitary fibrous tumour of the anterior thoracic wall.

On physical examination, the tumour appeared as an exophytic pseudolobulated tan-pink mass, measuring 18 × 10 cm, with multiple hemorrhagic foci, yellow and black necrosis, and localized infection with purulent exudates (Figure 1). There was no evidence of regional lymph node involvement. Laboratory examination revealed hypochromic microcytic anemia with hemoglobin serum level of 5.2 mg/dl, probably due to the intermittent bleeding of the tumour. The iron-deficiency anemia was relieved with the administration of 2 blood units. Magnetic Resonance Imaging (MRI) studies revealed no bone or cartilaginous involvement, apart from the chest wall soft tissue infiltration. The additional studies, including chest X-ray and abdominal ultrasound that were performed, were unremarkable with no evidence of metastatic disease. 

**Figure 2:**
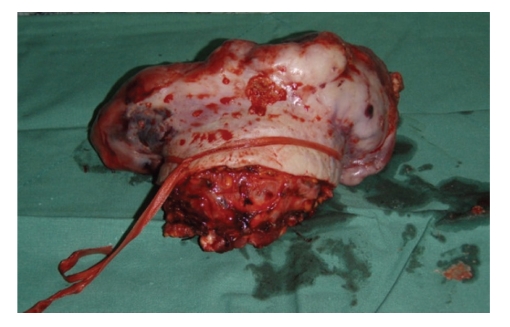
Tumour specimen after resection.

**Figure 3:**
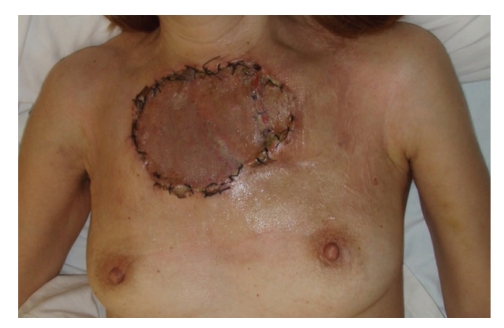
Immediate postoperative result after tumour resection and reconstruction with bilateral pectoralis major advancement flaps and split-thickness skin graft.

Because of the high vascularity of the tumour prior to incisional biopsy, a preoperative selective embolization of the right internal thoracica artery was performed in order to reduce vascularity and minimize intraoperative hemorrhage. The patient underwent wide local resection of the tumour by a surgical team consisting of thoracic and plastic surgeons (Figure 2). For the reconstruction of the wide defect both pectoralis major advancement flaps were mobilized and covered with a partial-thickness skin graft (Figure 3). 

**Figure 4:**
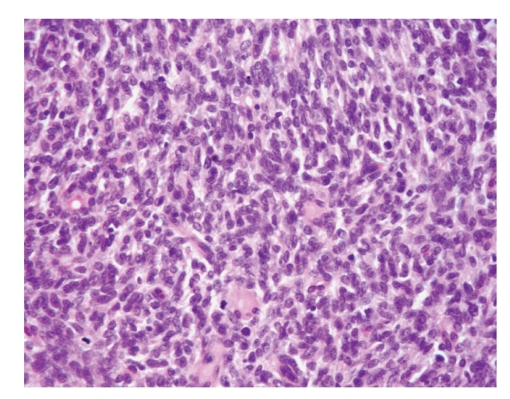
Malignant solitary fibrous tumour demonstrating patternless arrangement of spindle cells and areas with very high cellularity. The spindle cells have moderate cytological atypia, and three mitoses in mitotically active fields are evident (HE x400).

**Figure 5:**
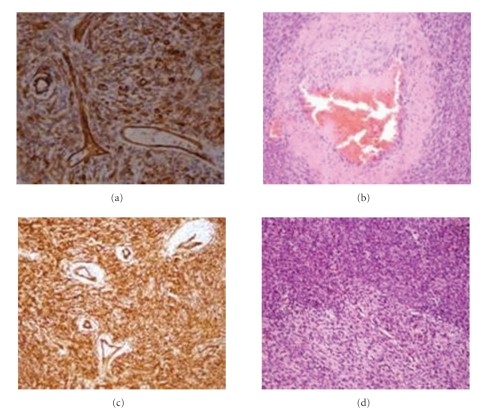
(a) Malignant solitary fibrous tumour demonstrating uniform immunoreactivity for CD34 (CD34 x400) with hemangiopericytoma-like morphology. (b) A hyalinized vessel with thrombus in it is evident (HE x200), as well as (c) hyalinized staghorn vessels of tumour, that are centered (CD34 x200). (d) Areas of hypo- and hypercellularity (HE x200) are evident.

The histological examination of the biopsy and resection specimen revealed a spindle cell tumour with strong diffuse CD34, Bcl-2, and vimentin positivity but negativity for S-100, c-kit, smooth muscle actin, and cytokeratin (AE1 + AE3). Immunohistochemically, the tumour cells were stained also negative with CD99. Microscopically spindle cells were arranged patternless, with a characteristic hemangiopericytoma-like morphology, and there were areas with very high cellularity (Figures 4 and 5). The spindle cells had moderate cytologic atypia and 9 mitoses per 10 HPFs. Foci of superficial necrosis were also identified (coagulative tumour necrosis). The tumour appeared centered on subcutaneous tissues. Surgical resection margins were not involved. The diagnosis of histologically malignant extrapleural SFT was confirmed. The differential diagnosis of this tumour, in particular, exclusion of a DFSP— tumour's original diagnosis—was made on the basis of its characteristic microscopic appearance in conjunction with immunohistochemical features. Histologically, SFTs present a typical, although not diagnostic, hemangiopericytoma-like morphology with patternless arrangement of spindle cells in a collagenous background whereas DFSPs are characterized mostly by a storiform pattern. In addition, DFSP stains frequently positive for CD34 but negative for Bcl-2.

Postoperatively the patient received adjuvant radiotherapy (Intensity-Modulated Radiation Therapy) and was closely followed up. In the ensuing 12 months after surgery and radiation treatment, the patient has remained asymptomatic and without clinical or radiological evidence of recurrence or distant metastasis.

## 3. Discussion

SFT is an uncommon neoplasm of mesenchymal origin that was first recognized by Klemperer and Rabin as a distinctive pleural lesion in 1931 [8]. Over the last 10 years, at least 106 extrapleural SFTs have been reported, located in the meninges, orbit, upper respiratory tract, salivary glands, thyroid, peritoneum, liver, retroperitoneum, adrenal gland, kidney, spermatic cord, urinary bladder and prostate spinal cord, periosteum, and soft tissue mainly in the head and neck [1–7, 9–15]. About 10%–36% of intrathoracic SFTs are histologically or clinically malignant while malignant SFTs have been described in 11%–33% of cases at other locations [2, 3]. To the best of our knowledge, this is the first case report of a histologically malignant extrapleural SFT located on the anterior thoracic wall.

Because of the overlapping of histological diagnosis of SFT with other soft tissue tumours, its correct and precise pathological characterization necessitates experienced soft tissue pathologists to evaluate the specimen both for a proper diagnosis as well as for the detection of malignant features. The differential diagnosis is based on the microscopical morphology (WHO 2003 criteria: patternless growth pattern, composed of round to spindle-shaped fibroblastic cells set in a collagenous matrix, hemangiopericytoma-like vasculature pattern often hyalinized thickened vessel walls) and characteristic immunohistochemical findings -positivity for CD34, Bcl-2, CD99, and vimentin [11]. The differential diagnosis is extensive including hemangiopericytoma, synovial sarcoma, dermatofibrosarcoma protuberans, leiomyosarcoma, liposarcoma, and malignant peripheral nerve sheath tumour [10, 11].

SFTs may present malignant behavior with local recurrence or metastasis, and there exist well-established pathological criteria of malignancy [14]. Vallat-Decouvelaere et al. [2] suggested atypical histological features, such as nuclear atypia, areas of increased cellularity, necrosis, and 4 or more mitoses per 10 high-power microscopic fields, as being predictive for clinical malignant behavior of the tumour and found local or distant relapse in those cases in 80%. In addition, according to Gold et al. [1] primary tumour size of more than 10 cm and positive surgical resection margins are positively correlated with unfavorable clinical outcome. In the studies of Vallat-Decouvelaere et al. [2] and Gold et al. [1] local recurrence appears in 4.3% and 6.7% and metastasis in 5.4% and 5.3%, respectively. Local recurrence or metastasis occurs most often within the first 2 years, while the sites of distant metastasis are most commonly the lung and the liver. Occasionally benign SFTs behave in a clinically malignant fashion particularly after a long period of time from the original tumour [1]. A more recent large series of extrapleural SFTs (ESFTs) with a long follow-up period reported by Cranshaw et al. [7] showed that ESFTs behave clinically in a manner similar to high-grade soft tissue sarcoma with relatively high rates of local recurrence (16.2%) and metastatic spread (8.9%) as well as with a poor overall prognosis (5-year survival rate 40%). Although presence of malignant histopathological features remains an indicator of a poor clinical outcome for these cases, no ESFT should be considered definitely benign currently. It should be emphasized that the fact that histologic appearance does not correlate perfectly with the clinical behavior in SFT is a fact of major clinical importance. Occasionally, histologically benign ESFTs are clinically malignant while many histologically malignant ESFTs are clinically benign. Therefore, extended follow-up is necessary to identify and manage the relatively high rate of recurrent disease [7]. It is clear that in the case of the presented patient, incomplete primary resections accounted for early locoregional recurrence. 

The treatment of choice for extrapleural SFT that is widely accepted is complete surgical extirpation with disease-free margins. Adjuvant radiation therapy and chemotherapy may be used especially in malignant variants of the disease or incomplete resections with no further surgical options. Due to the late recurrence or metastasis that may appear in the case of an SFT, an extended follow-up surveillance should be advised [1, 2, 11].

## 4. Conclusion

The behavior of SFT is unpredictable [14]. The risk of local recurrence and metastasis is high even in so-called “benign” tumours after a long period of time. Tumour specimens should be evaluated by experienced soft tissue pathologists. The treatment of choice is complete resection followed by extended follow-up surveillance [11].
